# A novel combined approach: trans-nasal endoscopic and transoral robotic-assisted resection of nasopharyngeal amyloidosis

**DOI:** 10.1093/jscr/rjag392

**Published:** 2026-06-14

**Authors:** Ishita Sen, Zaid Awad

**Affiliations:** Department of Head and Neck Surgery, Imperial College London, Imperial College Healthcare NHS Trust, Fulham Palace Road, London W6 8RF, United Kingdom; Department of Head and Neck Surgery, Imperial College London, Imperial College Healthcare NHS Trust, Fulham Palace Road, London W6 8RF, United Kingdom

**Keywords:** TORS, nasopharynx, amyloidosis

## Abstract

Amyloidosis is a condition characterized by extracellular deposition of insoluble, proteinaceous material with β-pleated ultrastructure. Localized head neck amyloidosis is rare with 41 cases of nasopharyngeal amyloidosis reported. A 56-year old male, with a history of T2 N1 M0 HPV positive left tonsillar squamous cell carcinoma, under surveillance post-treatment, had an incidental finding of a nasopharyngeal amyloidosis (AL lambda), involving the right lateral wall. This was resected with combined trans-nasal and transoral robotic-assisted surgery (TORS) approach, avoiding palatal split while using suction catheter for soft palate retraction. This is the first case of nasopharyngeal amyloidosis treated with this novel technique. The patient had complete resolution of swallowing with good palatal function by 2 months post-procedure and a 1 year recurrence free survival. TORS is a great adjunct for nasopharyngeal resections where conventional methods maybe limited. The combined approach added excellent 3D visualization and precise dissection without the sequel of palatal splitting.

## Introduction

Amyloidosis is widely known as a protein folding disorder characterized by the extracellular deposition of insoluble, amorphous, and proteinaceous material with a well-defined β-pleated sheet ultrastructure [1, 2]. It can be systemic or localized with the latter having a better prognosis. Localized amyloidomas of the head and neck are rare with the larynx being the commonest, followed by thyroid gland, trachea, and orbit. Only 41 cases of nasopharyngeal amyloidosis are reported in the literature with 23, surgically treated including laser resection. Two patients received adjuvant chemotherapy and one received postoperative radiotherapy [3].

In this case report, we describe a 56-year-old gentleman with nasopharyngeal amyloidosis, treated with combined trans-nasal endoscopic and transoral robotic-assisted surgery (TORS). This is the first reported case of nasopharyngeal amyloidosis treated with this novel technique.

TORS is routinely used for oropharyngeal cases and is being increasingly used for nasopharyngeal resections. The three-dimensional camera of the robot provides excellent visualization with the versatile Endowrists of the robotic arms, allowing navigation through narrow spaces and dissection around anatomical structures. With this combined approach, the Trans-nasal endoscopy helped achieve superolateral access, while, TORS with a 30° north facing angle, allowed approaching the nasopharyngeal region from behind the soft palate while minimizing tissue displacement and achieving a 3D image of the operative field [4–6].

## Case report

This 56-year-old gentleman with a T2 N1 M0 HPV positive left tonsil squamous cell carcinoma involving the left soft palate was treated with chemo-radiotherapy and bilateral nodal irradiation followed by left selective neck dissection for partial response on FDG PET CT scan.

**Figure 1:**
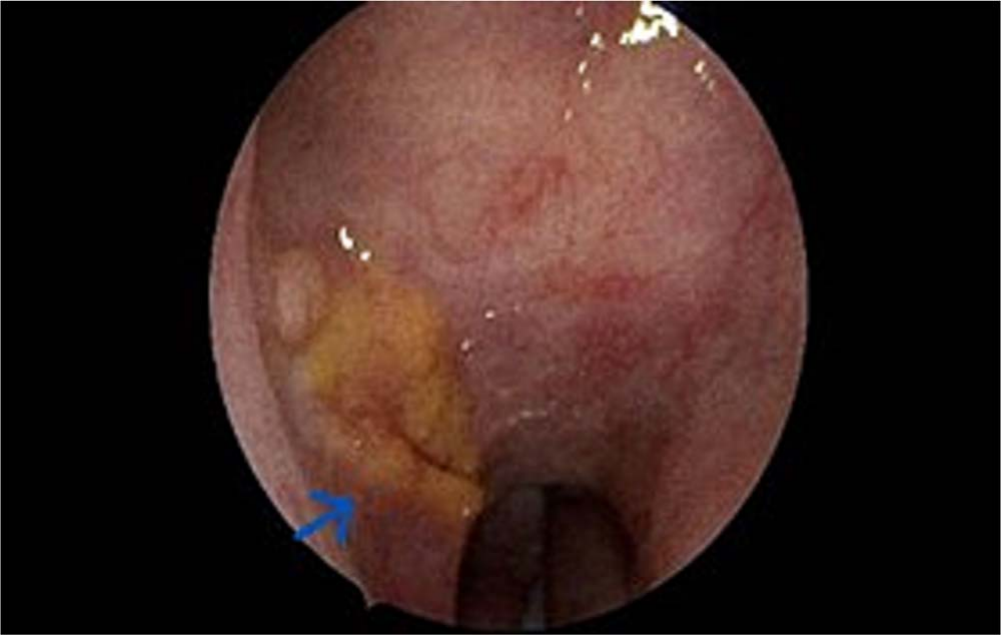
Trans-nasal endoscopic view (standard).

Routine clinic surveillance within a month following the last procedure revealed a new yellowish lesion involving the right lateral wall of the nasopharynx, in the postnasal space on flexible nasal endoscopic evaluation and a biopsy was taken due to high suspicion of a second primary (Fig. 1). The biopsy showed fragments lined by respiratory epithelium and underlying seromucinous glands with amorphous pink hypocellular nodules showing apple green birefringence on polarization with Congo.

Red staining which was also subjected to immunochemistry and proteomic analysis confirming, AL amyloid. A CT neck, thorax, and abdomen ruled out any evidence of cancer recurrence or systemic amyloidosis while establishing isolated nasopharyngeal amyloidosis. Clinical evaluation confirmed the local extent of the nasopharyngeal lesion, involving the right nasopharynx and extending into the retropalatal region of the oropharynx.

**Figure 2:**
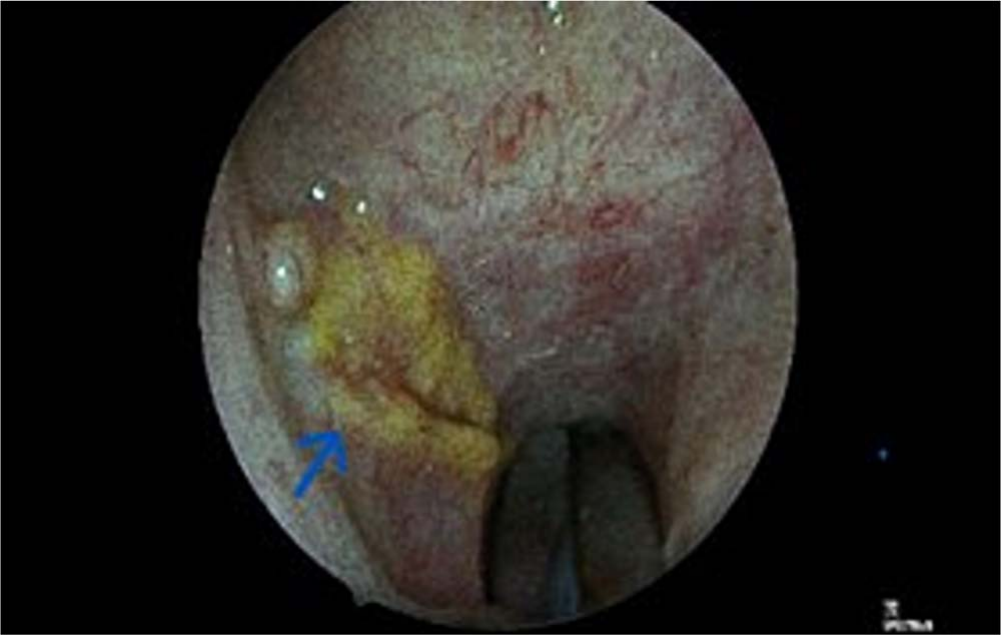
Trans-nasal endoscopic view (spectra B—narrow band image).

**Figure 3:**
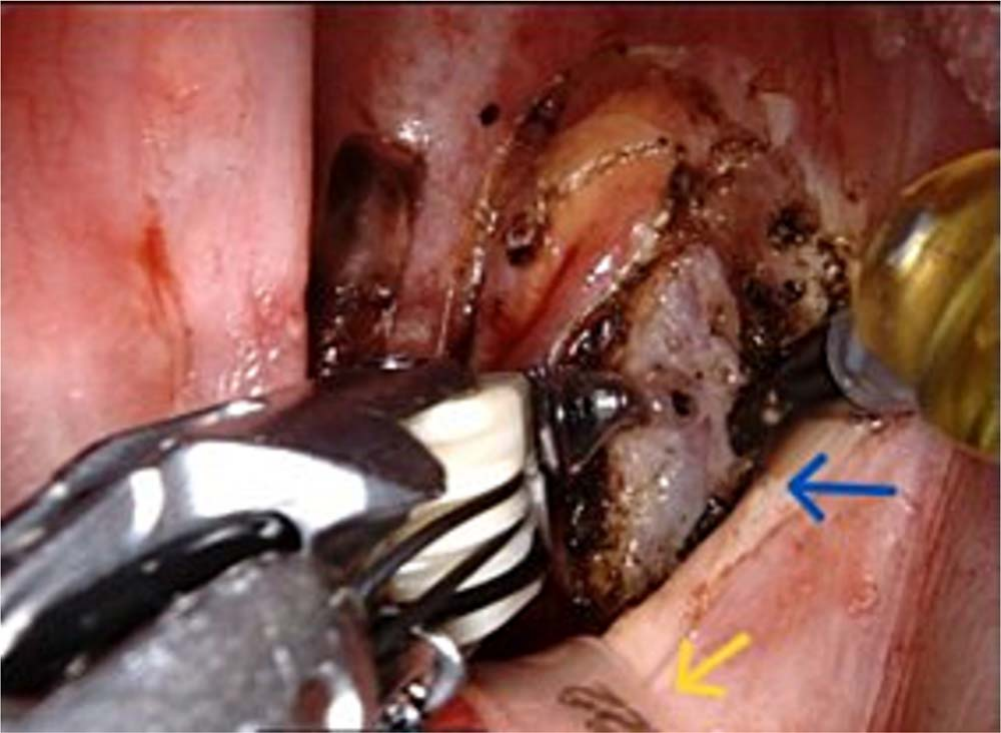
Transoral robotic resection (TORS) image, yellow pointer showing the suction catheter used to retract the soft palate and the blue pointer points towards the retracted soft palate and the retropalatal amyloidoma.

We performed a combined approach trans-nasal endoscopic-assisted and TORS right partial pharyngectomy. A good exposure was obtained with an endoscopic trans-nasal approach using 30° down facing telescope (Figs 1 and 2) and a Boyle-Davis mouth gag for TORS approach with upward facing 30° telescope. Cophenylcaine spray in the nose and Lignospan injected around the tumour helped reduce intraoperative bleeding. 1 g tranexamic acid, 1.2 g Co-amoxiclav, and 6.6 mg dexamethasone were given intravenously at the start of the surgery. The soft palate was retracted anteriorly with flexible suction catheters, in a similar fashion used for adenoid surgery (Fig. 3).

### Technical details

**Figure 4:**
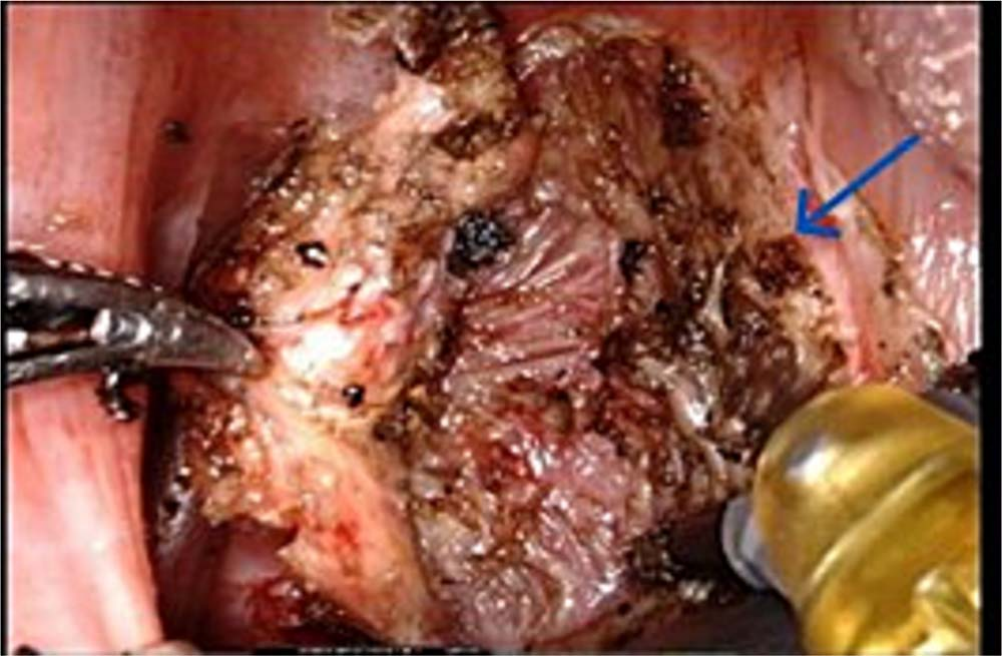
Transoral robotic resection (TORS) image showing the tumour being resected off the underlying superior constrictor muscle.

Trans-nasal endoscopic-assisted resection allowed release of the superior extent using monopolar diathermy away from and sparing the eustachian tube orifice. TORS, using the Da-Vinci Xi system (Intuitive Surgical) and its articulated arms with Maryland’s Bipolar Diathermy and Monopolar Diathermy forceps, the remainder of the amyloid mass was dissected carefully off the constrictor muscle and removed en-bloc trans-orally (Fig. 4). The resection cavity was washed, haemostasis achieved and absorbable haemostat (PuraBond) applied.

### Postoperative advice

The patient was started on regular NeilMed sinus rinse thrice a day through the right nostril for 5 days, Co-amoxiclav 625 mg orally, tranexamic acid 1 g thrice a day and Difflam mouthwash 4 times a day to gargle with, for 2 weeks. After a swallow assessment by the speech and language therapists, he was started on soft diet and was kept admitted to the hospital for a few days due to high bleeding risk. Final Histology and Immunohistochemistry: The findings were consistent with Amyloid of AL (lambda subtype). The margins were close but adequate. A wide margin was not required owing to the benign pathology.

### Follow up

During the first postoperative follow up, 3 weeks after, the patient had normal palatal movement and eustachian tube function with some swallowing difficulties and mild regurgitation with liquids on drinking quickly. A postoperative Barium swallow ruled out a stricture and showed some residual food bolus which was attributed mostly to weakened constrictors and dryness from previous radiotherapy for which he was advised swallowing therapy. These symptoms had resolved completely during the next follow up 2 months later, with significant improvement in the patient’s quality of life. The nasopharynx and oropharynx were clear on endoscopic evaluation. Since then, the patient has been on a 2 monthly follow up for 1 year with no signs of recurrence.

## Discussion

The nasopharyngeal amyloidosis cases treated surgically as described in literature, used endoscopic trans-nasal, combined oral-nasal approach or transoral approach, three of which advocated the use of plasma knife and one used CO_2_ laser. Though palate splitting was advocated in a small number these cases, whether any of these patients had previous history of irradiation has not been clearly mentioned [3]. This makes our case, the first case of nasopharyngeal amyloidosis where the use of TORS was advocated and treated with this novel technique allowing intricate dissection and adequate margins with minimal functional morbidity.

The combined technique of tran-nasal endoscopic and TORS has been advocated on few occasions for nasopharyngeal resections. Table 1 provides an overview. In all of these scenarios, an endonasal endoscopic approach was used with the two robotic arms inserted transorally and the soft palate split for access [6–9].

**Table 1:** An overview of nasopharyngeal resections done with the combined tran-nasal endoscopic and TORS approach.

1.	2010	Tsang *et al*.	Nasopharyngeal carcinoma
2.	2012	Dallan *et al*.	Skull base tumour
3.	2013	Carrau *et al*.	a. Nasopharyngeal and skull base malignancies (Cadaveric study) b. Adenoid cystic carcinoma with involvement of the middle cranial fossa, nasopharynx, clivus, and sphenoid sinus (Clinical Study)
4.	2014	Sreenath *et al*.	a. Metastatic Retropharyngeal mass in Papillary Thyroid Carcinoma b. Nasopharyngeal Melanoma c. Nasopharyngeal scarring
5.	2022	Han *et al*.	Recurrent Nasopharyngeal carcinoma (13 cases between 2017 and 2020)
6.	2025	Wagner *et al*.	Nasopharyngeal scarring

Dallan *et al.* in 2012 presented a modified combined approach performed with a central, endoscopic robotic arm in the nasal cavity and 2 robotic arms inserted through small, transcervical paramandibular windows [6]. This could be attributed to the old Si system with 5 mm robotic arms compared to the new da Vinci surgical system with 8 mm arms, unsuitable for trans-nasal surgery. The da Vinci Single Port (SP) system with 6 mm articulating arms is an alternate option however, has limited availability.

The exposure of the nasopharynx is increased by dissection of the muscular insertions of the soft palate from the hard palate [10]. This can compromise the nerve supply to the soft palate, cause impairment of pharyngeal constrictors, scarring, or stricture formation contributing to swallowing problems, including persistent dysphagia and velopharyngeal insufficiency.

In our case report, a procedure with least impact on function was agreed upon, considering the high dose radiotherapy to the left pharynx. The division of soft palate was avoided and a suction catheter used instead, to retract the soft palate anteriorly and optimize the view, which adds novelty to our technique. This prevented potential palsy of the right palate, risk of delayed healing, and fistula formation and gastrostomy tube dependency.

This novel, combined approach trans-nasal endoscopic and transoral robotic-assisted resection, thus, helped optimize result while minimally affecting patient’s quality of life.
